# Correction: Liao et al. Involvement of IL-1β-Mediated Necroptosis in Neurodevelopment Impairment after Neonatal Sepsis in Rats. *Int. J. Mol. Sci.* 2023, *24*, 14693

**DOI:** 10.3390/ijms26178513

**Published:** 2025-09-02

**Authors:** Zhimin Liao, Qing Zhu, Han Huang

**Affiliations:** Department of Anesthesiology and Key Laboratory of Birth Defects and Related Diseases of Women and Children, West China Second University Hospital of Sichuan University, Chengdu 610041, China; zhiminliao1982@163.com (Z.L.); zq82923@hotmail.com (Q.Z.)

In the original publication [1], there was a mistake in Figures 2C and 8F as published. In Figure 2C, the trace for group LPS was incorrect as it was the same as that in group Con. In Figure 8F, the representative blotting band for MLKL was incorrectly selected, which belongs to another publication from us [2] (cited as reference 42 in this IJMS publication). We were preparing these two original manuscripts at the same time, and these two sets of bands were of high similarity. We apologize for these unintentional errors. The corrected Figure 2C and Figure 8F appear below. The authors state that the scientific conclusions are unaffected. This correction was approved by the Academic Editor. The original publication has also been updated.

## Figures and Tables

**Figure 2:**
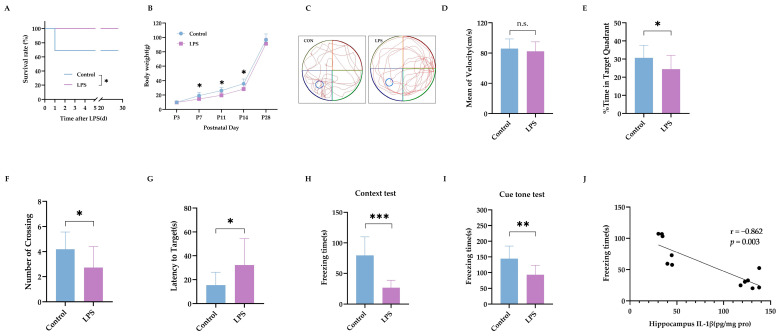
Intraperitoneal LPS injection on P3 led to long-term cognitive impairment. (**A**) The survival rates of rat pups following LPS injection (*n* = 16). (**B**) The increase in body weight in rat pups (*n* = 16 in the control group, *n* = 11 in the LPS group). (**C**) Representative traces of the MWM test. (**D**) The mean velocity of swimming during the MWM test. (**E**) Percentage of time spent in the target quadrant. (**F**) Number of platform crossings. (**G**) Latency time to find the area for the platform (*n* = 16 in the control group, *n* = 11 in the LPS group for (**D**–**G**)). (**H**) The freezing time of rats in the context test. (**I**) The freezing time of rats in the cue tone FC test (*n* = 16 in the control group, *n* = 11 in the LPS group for (**H**,**I**)). (**J**) The correlation between hippocampal IL-1β level and freezing time of context test (*n* = 6). LPS: lipopolysaccharide; MWM: Morris water maze; FC: fear conditioning. Data are expressed as the mean ± SD. * *p* < 0.05, ** *p* < 0.01, *** *p* < 0.001, n.s.: no significance.

**Figure 8:**
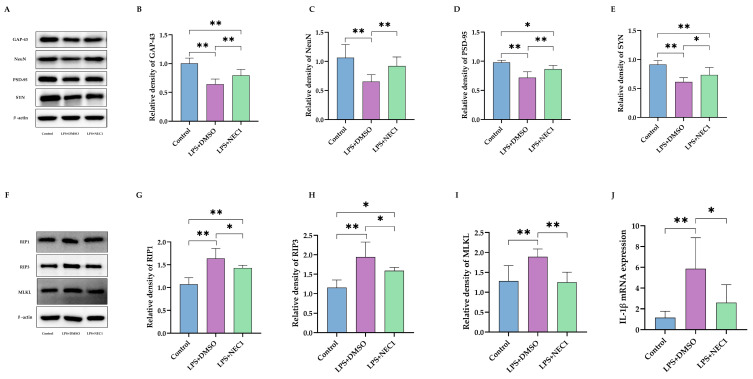
Inhibiting necroptosis reduced impairment of neuron development and synaptic function after neonatal LPS injection. (**A**) Western blotting with statistics for expression of GAP-43 (**B**), NeuN (**C**), PSD-95 (**D**), and SYN (**E**) (*n* = 6). (**F**) Western blotting with statistics for expression of RIP1 (**G**), RIP3 (**H**), and MLKL (**I**) (*n* = 6). (**J**) IL-1β mRNA expression in hippocampus after NEC1 treatment (*n* = 6). LPS: lipopolysaccharide; DMSO: dimethyl sulfoxide; NEC1: Necrostatin-1. Data are expressed as the mean ± SD. * *p* < 0.05, ** *p* < 0.01.
